# Scorpion species distribution and sting epidemiology in Dena and Boyer-Ahmad, Iran – Implications for management

**DOI:** 10.1016/j.parepi.2025.e00462

**Published:** 2025-10-02

**Authors:** Mohsen Fakhraei, Raziyeh Shahheidari, Amir Hossein Roozitalab, Kourosh Azizi, Mohsen Kalantari

**Affiliations:** a Student research committee, Department of Biology and Control of Disease Vectors, School of Health, Shiraz University of Medical Sciences, Shiraz, Iran; b Reseach Center for Health Sciences, Institute of Health, Department of Biology and Control of Disease Vectors, School of Health, Shiraz University of Medical Science, Shiraz, Iran

**Keywords:** Scorpion sting, Epidemiology, Dena city, Boyerahmad city, Iran

## Abstract

Scorpion envenomation represents a significant public health burden in Iran, particularly in Kohgiluyeh and Boyer-Ahmad Province, which reports 40,000 to 50,000 cases annually. The ecological adaptability and diversity of scorpions contribute to their widespread distribution, heightening the risk of human encounters and the associated economic and psychosocial impacts. This epidemiological study investigated scorpion distribution and sting patterns in Dena and Boyer-Ahmad counties, southwestern Iran, during a six-month sampling period from spring to September 2024. Specimens were collected diurnally and nocturnally using UV light within 500-m radii of predetermined sites, primarily from beneath rocks and old walls in desert environments. Among 208 identified scorpions, *Hottentotta zagrosensis* (*n* = 132) predominated, followed by *Hemiscorpius lepturus* (*n* = 28), *Compsobuthus rugosulus* (*n* = 24), *Mesobuthus eupeus* (*n* = 20), and *Orthochirus sp.* (*n* = 4). The Buthidae family accounted for 86.53 % of specimens. Concurrently, clinical and demographic data from 156 sting cases recorded in local health centers during 2024 were analyzed. Males comprised 54.5 % of cases, with hands (48.1 %) and feet (37.2 %) being the most frequent sting sites. Most incidents occurred indoors (82.7 %) and between 6 p.m. and midnight (31.4 %). Notably, no fatalities were reported. This research provides the first comprehensive faunistic and epidemiological data for these counties, revealing *H. zagrosensis* as the dominant species. The findings establish a critical foundation for developing targeted ecological management strategies, public health interventions, and conservation efforts to mitigate scorpion-related risks in southwestern Iran.

## Highlights

• **208 scorpion specimens were identified across 5 species in a** cross-sectional **survey** in Iran's Dena and Boyer-Ahmad counties.
• *Hottentotta zagrosensis* **dominates (63.5 %)**, challenging expected medically significant species prevalence.
• **156 sting cases show key risks:** Males (54.5 %), **hands (48.1 %)**, and **indoor settings (82.7 %)**, peaking 6 PM-midnight.
• **High indoor sting rate (82.7 %)** reveals significant human-scorpion cohabitation risks.
• **Critical baseline data** for targeted scorpion management and prevention strategies.

## 1 Introduction

There is approximately 2612 species of scorpions in the world. In Iran, there are four families: Buthidae, which includes 17 genera and 71 species; Hemiscorpiidae, which consists of one genus and seven species; and Scorpionidae, which has one species and two subspecies. This amounts to 19 genera, 96 species, and two subspecies (Bavani et al., 2022; Bardaran et al., 2024; Mahdi Kazemi et al., 2024). Iran boasts a rich array of climatic conditions and biodiversity, making it an ideal breeding ground for a wide variety of animals, including several species of scorpions. Previous research has identified temperature and rainfall as the most significant environmental factors influencing the distribution of scorpions. Furthermore, scorpions have a long evolutionary history, dating back 450 million years to the Silurian period (Lourenço, 2018; Lourenço and Cuéllar, 1995). Their diversity and adaptability to various environmental conditions have contributed to their spread across different geographical areas, consequently increasing the risk of scorpion stings among human populations. While scorpions are commonly associated with desert environments, they can also be found in forests, mountains, and under rocks, and they primarily feed on insects, spiders, and small arthropods (Sharifinia et al., 2017).

In Iran, approximately 51 species of scorpions have been documented, of which 11 are considered medically significant (Sanaei-Zadeh et al., 2017). Among the Iranian scorpion fauna, six species are classified as highly venomous: *Androctonus (A.) crassicauda*, *Hemiscorpius (H.) lepturus*, *Hottentotta (H.) saulcyi*, *H. zagrosensis*, *Mesobuthus (M.) eupeus*, and *Odontobuthus (O.) doriae* (Bavani et al., 2017; Rafinejad et al., 2020). In regions such as Khuzestan, Hormozgan, Bushehr, Sistan and Baluchistan, Kohgiluyeh and Boyer Ahmad, and Fars provinces, numerous scorpion species can sting humans, posing public health risks.

Scorpion stings are a significant health problem in many parts of the world, including Iran. These ancient arthropods have specialized mechanisms for injecting venom, which poses considerable health risks, especially in developing regions (Ebrahimi et al., 2020). In addition to Iran, scorpion stings are a major health problem in neighboring countries such as Oman, Iraq, Saudi Arabia, Pakistan, Yemen, and the United Arab Emirates. Furthermore, Iran has one of the highest rates of scorpion stings in the world, with 40,000 to 50,000 cases of scorpion stings occurring annually, and approximately 19 deaths per year (Rostampour et al., 2024). Furthermore, the mortality rate from scorpion stings in Iran is approximately ten times greater than that from snakebites. This represents a significant health issue related to other dangerous animals in Iran. The number of human deaths associated with scorpion stings is about one-third to one-half of those caused by snakebites (Firoozfar et al., 2019). Each year, scorpion stings result in significant economic costs as well as considerable psychological and emotional distress for society.

Clinical symptoms and mortality resulting from scorpion stings are influenced by two primary factors: the patient's condition, including age and overall health status, and the characteristics of the scorpion, such as species and venom potency. Most stings lead to localized pain. The most common symptoms and swelling, along with other manifestations such as changes in blood pressure, respiratory difficulties, excessive sweating, abnormal salivation, nausea and vomiting, dizziness, anxiety, seizures, confusion, renal symptoms, internal bleeding, abdominal pain, painful breathing, cold and damp extremities, thirst, fever, weakness, lethargy, palpitations, hypotension, restlessness, mental disturbances, and, in severe cases, death (Dehghani et al., 2016; Firoozfar et al., 2021; Howard et al., 2019; Khosravani et al., 2018; Shahi et al., 2009).

Iran, known for its high biodiversity, hosts various species of scorpions, particularly in the counties of Dena and Boyer-Ahmad, which offer favorable conditions for these creatures due to their unique geographical and climatic characteristics. Despite the ecological and biological significance of scorpions, occurrences of scorpion stings especially in rural and underdeveloped areas can pose serious public health concerns. Thus, this study focuses on determining the epidemiology of scorpion sting cases and identifying the scorpion fauna in the counties of Dena and Boyer-Ahmad in Kohgiluyeh and Boyer-Ahmad Province in 2024.

## 2 Methods & materials

### 2.1 Study area

The study conducted is a faunistic survey of a cross-sectional descriptive nature, focusing on scorpion species in selected areas of Dena and Boyer-Ahmad counties within Kohgiluyeh and Boyer-Ahmad Province.

Dena County is situated in the northern part of Kohgiluyeh and Boyer-Ahmad Province, with its administrative center located in the city of Sisakht. Dena County is bordered to the north by Lordegan County in Chaharmahal and Bakhtiari Province, to the east by Semirom County in Isfahan Province, to the south and southwest by Boyer-Ahmad County, and the west by Margoon County. Notable elevations in Dena County include Dena Peak, also known as Dinar Peak, which reaches an altitude of 4409 m, making it the ninth-highest peak in Iran. Each year, many climbers from across the country and around the world come to Sisakht to scale this peak and others in the area. According to the general population and housing census conducted in 2016, Dena County had a population of 42,539 people.

Boyerahmad County is one of the counties in Kohgiluyeh and Boyer-Ahmad Province, with the city of Yasuj serving as its administrative center. Boyerahmad County is bordered to the north by Dena and Margoon counties, to the northeast by Semirom County in Isfahan Province, to the west by Kohgiluyeh County, to the southwest by Charam County, to the south by Rostam and Mamassani Counties in Fars Province, and the east by Sepidan County in Fars Province. According to the general census of population and housing conducted in 2016, the population of Boyer-Ahmad County was 280,009 people.

**Fig. 1:**
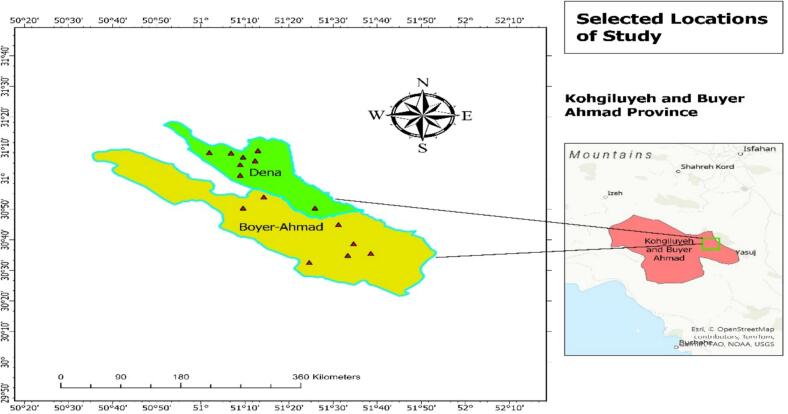
Sampling sites of scorpions in Dena and Boyer-Ahmad province, 2024.

In Dena county, 8 and in Boyer-Ahmad county, 7 sampling locations were randomly selected using cluster sampling, resulting in a total of 15 sampling locations (Fig. 1, Table 1). The map of the study location created using Arc GIS software (Price, 2020).The external locations were examined for the presence of scorpions, and ultimately, samples were collected. At each location, sampling was conducted within a radius of 500 m from the specified sites.

**Table 1:** Geographic coordinates of sample collection points.

Region name	Longitude	Latitude
Kale Gah	51.16629727587785	31.115083900870047
Rudashti	51.185182489268676	31.082367982446698
Dozdak2	51.17942584940163	31.089933392885285
Dozdak1	51.179935149037696	31.09199622801445
Sarsour	51.15210554704234	31.10832225135947
Banistan	51.23182021726147	31.085288217950204
Dorah	51.13424603070261	31.114261408563348
Bagh	51.238439386719605	31.006458582923788
Not reaching Si Sakht	51.384228673712336	30.894042822349984
Old Road of Shiraz	51.62773988085776	30.593514541738163
Dasht-e-Rum	51.552237185631895	30.560218120108715
Mukhtar-Kalos Road	51.50431621770877	30.673836172869258
Ganjai	51.56017998311393	30.738757664653402
Demchenar	51.2021377637873	30.862694505702247
Sarvuk	51.61815321342756	30.613583209006666

### 2.2 Ethical approval

This study was conducted in accordance with international, national, and institutional ethical guidelines. We declare that all experiments were performed in accordance with the ARRIVE guidelines 2.0 and that all experimental protocols were approved by Ethical approval obtained from the Science and Ethics Committee of Shiraz University of Medical Sciences (Approved ID: IR.SUMS.SCHEANUT.REC.1401.134).

### 2.3 Scorpion samplings

we conducted the collection and sampling of scorpions over six months, from early spring to the end of September 2024. Scorpions were collected from various locations at different times of day, and in some cases, solely at night using UV light method (Stahnke, 1972). Collection sites included peripheral areas beneath stones, as well as around old walls and in desert regions. Scorpions were captured using tweezers and pliers, occasionally with the assistance of ultraviolet light at night. The collected samples were preserved in 70 % alcohol and subsequently transported to the entomology laboratory of the Department of Biology and Vector Control at Shiraz University of Medical Sciences for identification. For identification, a pictorial key to Iranian scorpions, adapted from Farzan P and Dehghani, and other authoritative keys and expert colleagues in the field of specimen identification were used.

### 2.4 Scorpion sting data

Clinical and demographic data on scorpion stings were collected using an archive checklist from the Disease Control Center of the Dena and Boyer-Ahmad Health Departments for the year 2024. Demographic information including age, gender, occupation, and location of the patient's bite, among other things, was presented in Table 1, Table 2. Data analysis was conducted using SPSS27 software (Verma, 2012).

**Table 2:** Demographic Characteristics of Patient with Scorpion Sting.

Variables	Categories	Frequency	Percent
Sex			
	Male	85	54.5
	Female	71	45.5
Job			
	Housekeeper	50	32.1
	Self-employment	42	26.9
	Student	22	14.1
	Farmer	12	7.7
	Employee	6	3.8
	Other	24	15.4
The interval between sting and admission			
	Less than 2 h	149	95.5
	2–4 h	7	4.5

### 2.5 Human ethic notes

All subjects gave informed consent for inclusion before participating in the study. “Informed consent” was obtained from all subjects and/or their legal guardians.

## 3 Results

**Fig. 2:**
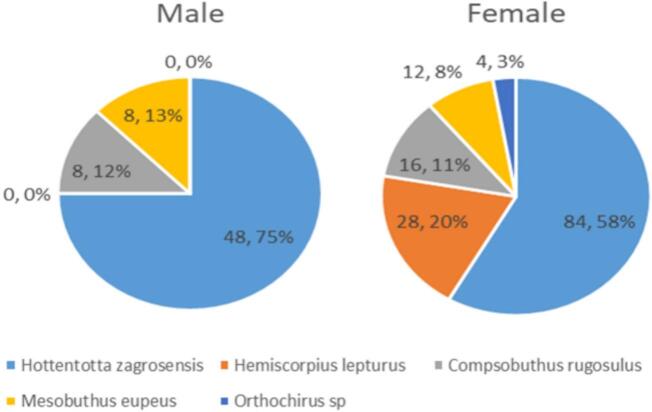
The percentage of male and female species of scorpions caught in Dena and Boyer Ahmad cities.

In this study, 208 scorpion specimens of different species including *Hemiscorpius lepturus*, *Hottentotta zagrosensis*, *Mesobuthus eupeus*, *Compsobuthus rugosulus*, and *Orthochirus sp* were identified in selected areas of Dena and Boyer-Ahmad counties*.* Buthidae (86.53 %) and Hemiscorpiidae (13.46 %) families were collected and identified*. Hottentotta zagrosensis* (*N* = 132) was the most abundant species in the study area, and *Orthochirus sp* (*N* = 4) was the least abundant species. The percentages of male (*N* = 64) and female (*N* = 144) scorpions were 30.76 % and 69.23 %, respectively (Fig. 2).

**Fig. 3:**
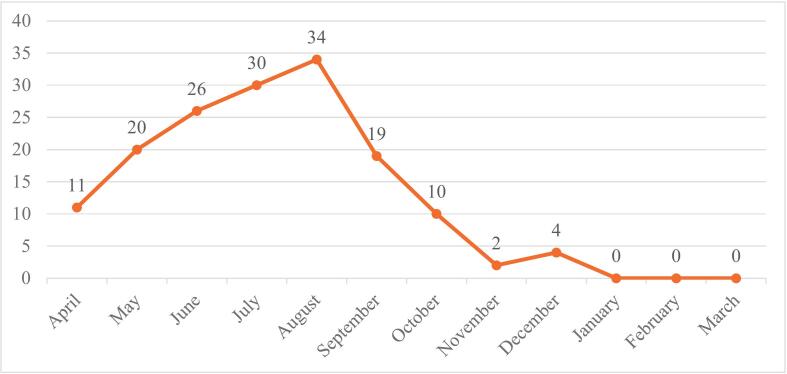
Distribution of Scorpion sting cases by month in Dena and Boyer-Ahmad cities during 2024.

All 156 cases of scorpion stings in the health centers of Dena and Boyer Ahmad counties admitted to the specified hospitals in 2024 were treated and subsequently discharged, with no reported fatalities resulting from scorpion stings (Fig. 3).

A total of 156 patients (54.5 %) who suffered from scorpion stings were male. The most common site of the sting was the hands, accounting for 48.1 % of cases, followed by the feet at 37.2 %. The head and neck had the lowest incidence of stings, at 6.4 %. The highest frequency of scorpion stings occurred between 6 p.m. and 12 a.m. (31.4 %). Additionally, 129 individuals (82.7 %) were stung indoors, while 27 individuals (17.3 %) were stung outdoors. It was also noted that 51.3 % of the scorpions were yellow except Gadim, 41.7 % were black, and 7.1 % were not specified. Table 2 presents the descriptive statistics for the participant's demographic characteristics (Table 2), and Table 3 the frequency distribution of clinical characteristics of Scorpion stings cases (Table 3).

**Table 3:** Frequency distribution of clinical characteristics of Scorpion stings cases.

Variables	Categories	Frequency	Percent
The anatomical area of the sting	Hands	75	48.1
	Legs	58	37.2
	Head and neck	10	6.4
	The middle part of the body	13	8.3
Location of case	Inside	129	82.7
	Outside	27	17.3
Time of sting	12 a.m. to 6 a.m.	35	22.4
	6 a.m. to 12 p.m.	32	20.5
	12 p.m. to 6 p.m.	40	25.6
	6 p.m. to 12 a.m.	49	31.4
Local symptoms of the patient when visiting the medical center	Numbness and muscle cramps	2	1.3
	Severe muscle pain	47	30.1
	Pain at the sting site	103	66.0
	Swelling and redness around the sting site	4	2.6
Scorpions	*Hottentotta zagrosensis*	65	41.7
	*Hemiscorpius lepturus*	80	51.3
	Not specified	11	7.1

## 4 Discussion

This research explored the bionomics and epidemiology of scorpions from the families Buthidae and Hemiscorpiidae in the Dena and Boyer-Ahmad regions of southwestern Iran, resulting in the collection of 208 specimens. Of the four scorpion families reported in Iran (Buthidae, Scorpionidae, Hemiscorpiidae, and Euscorpiidae) the Buthidae family has the highest frequency. The results of this study showed that the most abundant specimens collected in the study areas belonged to the species *Hottentotta zagrosensis.* This aligns with previous studies that demonstrate the diverse scorpion populations found in Iran's mountainous and dry regions (Rafinejad et al., 2020; Dehghani et al., 2016; Bavani et al., 2021; Motevalli Haghi and Dehghani, 2017). The characteristics of *H.zagrosensis* include its black color, its woolly body, and the equal size of its tail bands. Conversely, the genus Orthochirus had the fewest specimens, potentially due to its reduced adaptability or rarity in this habitat.

**Fig. 4:**
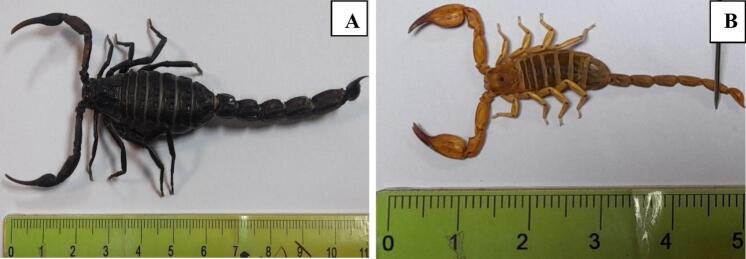
Scorpions *Hottentotta zagrosensis* (A) and *Hemiscorpius lepturus* (B).

In addition to the predominant species studied, *Hemiscorpius lepturus* also plays a significant role in the scorpion fauna of the Dena and Boyer-Ahmad regions. This species, belonging to the family Hemiscorpiidae, is known for its unique adaptations to the local environment. *H. lepturus* is typically characterized by its yellow color, slender body, and long legs, which aid in its ambush hunting behavior (Fig. 4). Most fatal scorpion envenomations in Iran are attributed to the species *H. lepturus* and *A. crassicauda* (Sanaei-Zadeh et al., 2017; Kazemi-Lomedasht et al., 2017). Hemiscorpius lepturus is a dangerous species that has been found in Iran, Oman, Iraq, Saudi Arabia, Yemen, Pakistan and United Arab Emirates. These scorpions are usually found in hot and humid areas. In Iran, they have been reported in the provinces of Khuzestan, Semnan, Fars, Kurdistan, Hormozgan, Sistan and Baluchestan, Isfahan, Bushehr, Kohgiluyeh and Boyer-Ahmad, Ilam, Chahar Mahal and Bakhtiari, Lorestan, Hamedan, Kermanshah, and Kerman (Dehghani et al., 2018).

Previous studies have documented its venomous nature, which can pose health risks to humans, particularly in rural areas where encounters are more frequent (Rafinejad et al., 2020; Dehghani et al., 2023). The histological results indicated that *H. lepturus* venom induced pathological changes in the kidneys, liver, skin, stomach, heart, spleen, lungs, small intestine, pancreas, bladder, and adrenal glands (Dehghani et al., 2023; Dehghani et al., 2012). The presence of *H. lepturus* in this region indicates that environmental conditions support its survival and reproductive success, contributing to the overall biodiversity of scorpions in southwestern Iran (Sanaei-Zadeh et al., 2017).

Another of the most abundant and important scorpion species in Iran is *Mesobutus opeus*, which has been caught in other regions of Iran in line with our study (Sharifinia et al., 2017; Bavani et al., 2017; Moradi et al., 2015). This species is widely distributed in Iran, and it has been reported in most studies of scorpion fauna, demonstrating greater locational and ecological diversity than other species. *M. eupeus*, being a non-digging species, has adapted well to living in close proximity to humans. There have been no reported deaths or stings associated with this species, suggesting that it is likely safe. Studies on the distribution of scorpion species in Iran indicate that *M. eupeus* is found in Gonabad, Hormozgan, Shiraz, and the Persian Gulf islands (Jamshidi et al., 2023).

Most specimens were collected from clay wall edges and beneath stones, typical scorpion habitats. These microhabitats offer favorable humidity and shelter from harsh conditions, indicating the scorpions' preference for such environments. Prior research has also shown that various environmental factors, including soil type and structure, influence scorpion habitat selection (Rafinejad et al., 2020).

In total, 156 cases of scorpion stings were reported in Dena and Boyer-Ahmad cities during 2024. Data from various studies indicate that most scorpion stings occur in men, as they are more likely to work in high-risk environments and, consequently, encounter scorpions more frequently. A study by Kassiri et al. in Masjed Soleiman showed that 50.5 % of scorpion stings occurred in men (Kassiri et al., 2019). Another study in 2018 in northern Khuzestan reported that 51.3 % of scorpion stings occurred in men (Kassiri, 2018). Furthermore, other studies reported an incidence rate of 60.25 % in Qom (Saghafipour et al., 2017), 62.44 % in Qeshm (Khosravani et al., 2018), 74 % in Isfahan (Gheshlaghi et al., 2010), 75 % in Oman (Al Abri et al., 2019), and 72 % in Qatar, in men (Alkahlout et al., 2015).

In the present study, most scorpion stings were reported in the hands and feet, and the lowest number of stings was found on the victims' head and neck. A study by Khosravani et al. in Qeshm showed that 44.68 % of the stings were in the hands, 42.35 % of the stings occurred in the legs, and only 5.35 % of the stings were found in the head and neck (Khosravani et al., 2018). A study by Kassiri in northern Khuzestan found most stings (39.7 %) on the legs.

The present study revealed that the majority of scorpion bites were inflicted by yellow scorpions. Research by Saghafipour et al. noted that 18.7 % of the victims were bitten by black scorpions (Saghafipour et al., 2017). The present study also revealed that most scorpion bites occur indoors. Many of these indoor stings can be attributed to the use of inappropriate building materials. Additionally, older buildings provide an ideal habitat for scorpions. Furthermore, sleeping on the floor instead of in a bed, placing blankets, sheets, and clothing on the ground which scorpions may use as shelter during the day failing to wear proper footwear, especially at night, and a lack of awareness regarding the dangers of scorpion stings and their symptoms can all contribute to an increased risk of scorpion stings indoors. Despite the presence of potentially dangerous species such as *Hemiscorpius lepturus* in the study area, no fatalities were reported during the study period. This outcome may be attributed to several contributing factors. First, the high rate of indoor stings may have led to quicker recognition and faster access to medical care. Also, the widespread availability of antivenom in local health centers and increased public awareness in the region might have played a preventive role.

## 5 Conclusions

Detailed biological and epidemiological surveys of scorpions in these areas have provided valuable information on their distribution patterns, habitats, and ecological behaviors. The results showed that environmental and human factors have a significant impact on the population and prevalence of scorpions. This research not only contributes to a better understanding of the biological diversity of these creatures but is also very important for the management and prevention of risks from their stings in local communities. Therefore, it is recommended that educational and information programs about scorpions and their associated risks be provided to residents of these areas to increase public awareness and minimize risks.

## CRediT authorship contribution statement

**Mohsen Fakhraei:** Writing – review & editing, Visualization, Formal analysis. **Raziyeh Shahheidari:** Writing – review & editing, Visualization, Formal analysis. **Amir Hossein Roozitalab:** Writing – review & editing, Visualization, Formal analysis. **Kourosh Azizi:** Writing – review & editing, Investigation, Data curation. **Mohsen Kalantari:** Writing – review & editing, Writing – original draft, Validation, Supervision, Methodology, Conceptualization.

## Consent to participate

Not applicable.

## Consent to publication

The authors fully consent to the publication of the article.

## Ethical statements

All subjects gave informed consent for inclusion before participating in the study. The protocol was approved by the Ethics Committee of Shiraz University of Medical Sciences (IR.SUMS.SCHEANUT.REC.1401.134) and the Research project code is 27005.

## Funding

This research was funded by the Shiraz University of Medical Sciences.

## Declaration of competing interest

The authors of the present study declare no conflict of interests.

## Data Availability

The data supporting the findings of this study are available within the article.
